# Effect of anesthesia on the outcome of high-grade glioma patients undergoing supratentorial resection: study protocol for a pragmatic randomized controlled trial

**DOI:** 10.1186/s13063-022-06716-9

**Published:** 2022-09-27

**Authors:** Jia Dong, Dexiang Wang, Huizhong Sun, Min Zeng, Xiaoyuan Liu, Xiang Yan, Ruowen Li, Shu Li, Yuming Peng

**Affiliations:** Department of Anesthesiology, Beijing Tiantan Hospital, Capital Medical University, Beijing, People’s Republic of China

**Keywords:** High-grade glioma, Anesthesia, Survival

## Abstract

**Background:**

High-grade glioma (HGG) is the most malignant brain tumor with poor outcomes. Whether anesthetic methods have an impact on the outcome of these patients is still unknown. Retrospective study has found no difference between intravenous and inhalation anesthesia on the overall survival (OS) of the HGG patients, however, intravenous anesthesia with propofol might be beneficial in a subgroup of patients with a Karnofsky Performance Status (KPS) Scale less than 80. Further prospective studies are needed to evaluate the results.

**Methods:**

This is a single-centered, randomized controlled, parallel-group trial. Three hundred forty-four patients with primary HGG for tumor resection will be randomly assigned to receive either intravenous anesthesia with propofol or inhalation anesthesia with sevoflurane. The primary outcome is the OS of the patients within 18 months. Secondary outcomes include progression-free survival (PFS), the numerical rating scale (NRS) of pain intensity and sleep quality, the postoperative encephaloedema volume, complications, and the length of hospital stay of the patients.

**Discussion:**

This is a randomized controlled trial to compare the effect of intravenous and inhalation anesthesia maintenance on the outcome of supratentorial HGG patients. The results will contribute to optimizing the anesthesia methods in these patients.

**Trial registration:**

ClinicalTrials.gov NCT02756312. Registered on 29 April 2016 and last updated on 9 Sep 2020

**Supplementary Information:**

The online version contains supplementary material available at 10.1186/s13063-022-06716-9.

## Background

High-grade glioma (HGG) accounts for 80% of malignant brain tumors. It is classified by the World Health Organization (WHO) as a grade III or IV tumor and develops the worst prognosis among brain tumors [1, 2]. WHO grade III refers to anaplastic gliomas, including anaplastic astrocytoma, anaplastic oligodendrocytoma, and anaplastic oligoastrocytoma. Most of WHO grade IV HGGs are glioblastomas. Most of HGG patients are glioblastoma patients, with a median overall survival (OS) of 16 months and progression-free survival (PFS) ranging from 7 to 10 months even under the optimized treatment [3, 4].

Up to now, surgery is still the first-choice treatment to reduce tumor burden and intracranial pressure (ICP). However, most of the inhalation anesthetics and the opioids during surgery were regarded as immunosuppressive and enhanced the invasiveness of the tumor cells by preclinical studies. The inhalation anesthetics were found to inhibit natural killer (NK) cell activity [5, 6], and upregulate the level of hypoxia-inducible factor (HIF), thus promoting tumor cell proliferation and metastasis, while intravenous anesthesia with propofol can reduce the level of HIF [7]. In vitro study has found that the expression of HIF increased after 6h exposure to 2% sevoflurane in astrocytes [8]. On the contrary, propofol promoted the programmed apoptosis of glioblastoma and astrocytoma cells [9, 10]. Propofol also took a protective anti-inflammatory effect by reducing the neutrophil–lymphocyte ratio (NLR) and platelet–lymphocyte ratio (PLR) of the patients [11]. All of these are expected to be further verified in the long-term outcome of the patients in prospective clinical studies.

In the retrospective study of 294 patients with primary supratentorial HGGs undergoing elective tumor resection, anesthesia maintenance with sevoflurane did not significantly worsen the PFS of the patients compared with propofol. However, sevoflurane might decrease the OS in patients with a Karnofsky Performance Status (KPS) scale of less than 80 [12]. The KPS scale is subjectively evaluated and recorded by a neurosurgeon preoperatively based on the following hierarchical scale: 100=normal, no evidence of disease; 90=able to perform normal activity with only minor symptoms; 80=normal activity with effort, some symptoms; 70=able to care for self but unable to do normal activities; 60=requires occasional assistance, care for most needs; 50=requires considerable assistance; 40=disabled, require special assistance; 30=severely disabled; 20=very sick, requires active supportive treatment; and 10=moribund.

The OS of patients with lower KPS were more influenced by the choice of anesthetics. Several reasons might be related to the results. Firstly, sevoflurane increases cerebral blood flow and decreases the cerebral metabolic rate by decoupling of the relationship between blood flow and oxygen consumption of the brain tissue. It increases ICP and reduces the compliance of brain tissue, which aggravates the neurofunction of the lower KPS patients [13]. However, propofol reduces cerebral blood flow and cerebral oxygen consumption simultaneously, which benefits for brain tumor patients with higher ICP. Secondly, compared with sevoflurane, propofol reduces the release of inflammatory factors and vascular permeability, which may reduce the severity of encephaloedema, and contribute to the complete resection of the tumor and the early recovery of patients [14]. Lastly, propofol improves postoperative analgesia and promotes rapid recovery compared with inhalational anesthetics [15, 16]. So propofol has been recommended for better ICP control and cerebral hemodynamics of patients in neurosurgical anesthesia [17, 18]. In the retrospective study, the lower KPS patients mainly included the aged, weak, disabled, or paralyzed patients. The anesthesia methods might affect the long-term outcome through aggravating the neurofunction and systematic condition of patients, however, their effects on tumor malignancy, invasiveness, and host cellular immunity have not been identified.

Although opioids are used perioperatively in common to block most of the pain or noxious stimulations, they also have a negative effect on the cellular immunity and outcome of the patients to some extent [19]. Local anesthetics by scalp infiltration or nerve block reduce the pain effectively, save the consumption of opioids or inhalation anesthetics, and improve the prognosis of HGG patients [20, 21]. However, in the previous retrospective studies, the pain intensity, depth of anesthesia, and consumption of the anesthetic or analgesic agents were not monitored or comparably controlled and might confound the results [22, 23].

Although some retrospective studies about other tumor/cancer patients have shown that anesthesia affects patients’ outcomes, it is still controversial. An analysis of 7030 patients found that inhalation anesthesia significantly increased the risk of death compared with intravenous anesthesia, with a hazard ratio of about 1.46 (1.29~1.66) [24]. Jun et al. found that inhalation anesthesia with isoflurane, sevoflurane or desflurane increased the risk of recurrence and death of patients compared with intravenous anesthesia in the esophageal cancer patients [25]. Propofol significantly improved the survival of colon cancer patients compared with desflurane anesthesia [26]; however, it did not improve the prognosis of non-small cell lung cancer patients compared with inhalation anesthesia [27]. The retrospective collection of clinical data, un-stratified American Society of Anesthesiologists (ASA) classification, tumor pathology, and surgery type are all limitations of those studies. A recent systematic review and meta-analysis summarized all previous studies by a subgroup of cancer sources and indicated that intravenous anesthesia had a certain advantage in general, but not sure in specific tumor source [28].

Therefore, we will test the hypothesis that intravenous anesthesia with propofol could increase the 18 months OS in HGG patients with KPS<80 undergoing tumor resection compared with sevoflurane inhalation anesthesia in this randomized-controlled trial.

## Methods and materials

### Study design

**Fig. 1:**
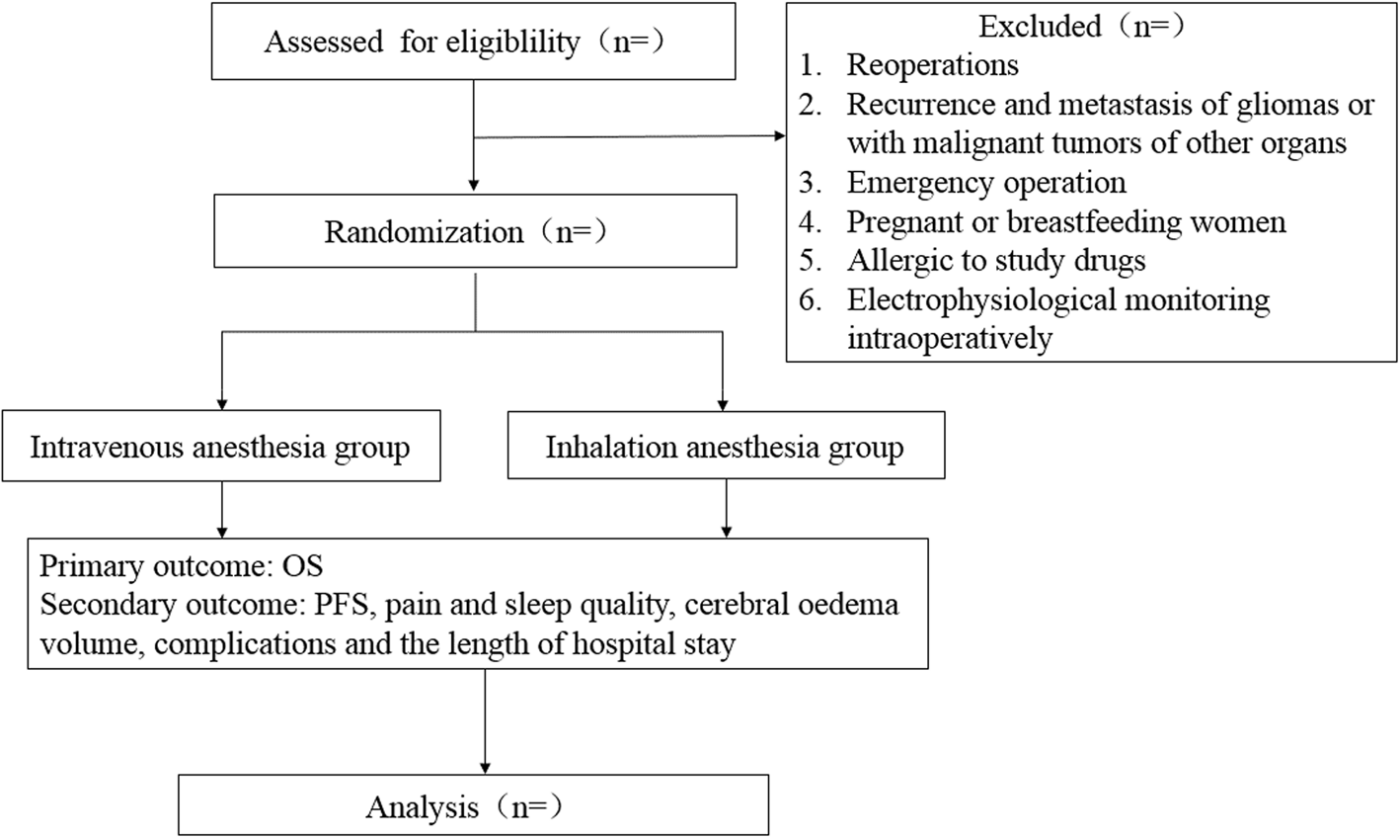
Consolidated standards of reporting trials flow diagram. OS, overall survival; PFS, progression-free survival

**Fig. 2:**
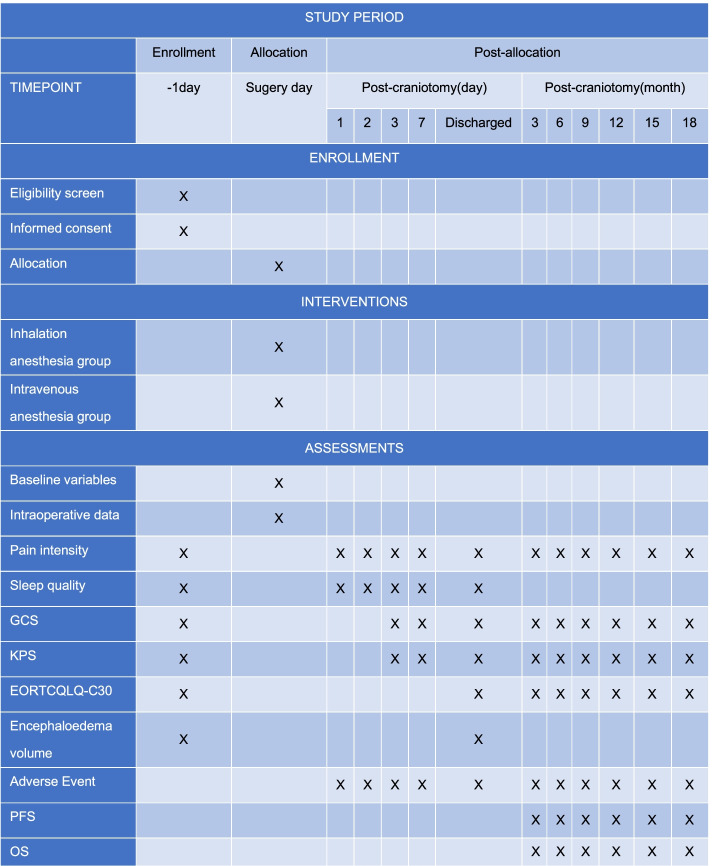
Standard protocol items: Recommendations for interventional trials. GCS, Glasgow Coma Scale; KPS, Karnofsky Performance Status; EORTCQLQ-C30, European organization for the research and treatment of cancer on quality of life; PFS, progression-free survival; OS, overall survival

This is a single-center, randomized-controlled, and paralleled-group trial being conducted at Beijing Tiantan Hospital, Capital Medical University (Fig. 1). The study was approved by the Ethics Review Committee of China Registered Clinical Trials on March 30, 2020 (number: ChiECRCT-20200049) and updated at www.clinicaltrials.gov on April 1, 2020 (number: NCT02756312). The schedule of enrollment and assessment is shown in the Standard Protocol Items: Recommendations for Interventional Trials (SPIRIT) (see Fig. 2). Written informed consent will be obtained from the legal representatives.

### Study population

#### Participants

Patients undergoing elective tumor resection under general anesthesia will be recruited consecutively from neurosurgical wards in Beijing Tiantan Hospital, Capital Medical University from Sept 2020 to December 2023.

Inclusion criteria include age more than 18 and less than 80 years old, preoperative KPS less than 80, and diagnosis of HGG by preoperative magnetic radiology imaging (MRI). The patients or their legal representatives agree to provide for the collection and use of participant data and biological specimens and sign written informed consent.

Exclusion criteria include a history of other operations after diagnosis of HGG, patients with recurrence and metastasis of gliomas or with malignant tumors of other organs, emergency surgery, pregnant or breastfeeding women, allergic to study drugs, patients who need electrophysiological monitoring during operation will be removed.

### Randomization and blinding

Block randomization will be conducted via computer-generated codes by an independent research assistant who will pack the allocation sequence with identical sequentially numbered opaque envelopes and distribute to the researcher according to the enrolling sequence. The independent research assistant should not recruit and allocate the participants for the purpose of avoiding selection bias.

Patients will be randomly assigned to the two groups at a 1:1 ratio. At the surgery morning, just before anesthesia induction, the trained attending anesthesiologists will receive the sealed envelope and know about the grouping. They will perform anesthesia strictly following the protocol and record intraoperative information. Trained follow-up personnel will not participate in the clinical anesthesia and postoperative management, or know the records of other researchers. The researcher assistant, patients, and outcome assessors will be blinded to the allocation, intraoperative management, or perioperative information until the completion of the study analysis unless specific circumstances, such as the occurrence of a serious adverse event (SAE).

### Intervention

The enrolled patients will be randomly assigned to the intravenous group or the inhalation group. Propofol will be used for anesthesia maintenance with the initial dose of 5–7mg/kg/h in the intravenous group. Sevoflurane will be used for anesthesia maintenance with an initial concentration of 2–3% in the inhalation group. During anesthesia, the same depth of anesthesia will be maintained by adjusting the dosage of propofol or sevoflurane, keeping bispectral index (BIS) range between 40 and 60. Inhalation of sevoflurane or infusion of propofol and remifentanil will be discontinued at the end of surgery. The adherence of intervention will be guaranteed by the common and routine method of anesthesia maintenance administered and intraoperative monitoring and recording in the trial.

### Concomitant treatment

Routine monitoring will include electrocardiographs, noninvasive blood pressure, pulse oxygen saturation, body temperature, and BIS. The BIS electrodes will be placed on the contralateral side of the tumor. The core temperature will be monitored continuously by an esophageal probe. Peripheral venous access will be established before anesthesia induction. Continuous arterial pressure, urine output, and end-tidal carbon dioxide partial pressure (PetCO_2_), minimal alveolar concentration (MAC) of inhalation agent will be monitored after anesthesia induction. All patients will be premedicated with midazolam (0.05 mg/kg) intravenously 5 min before anesthesia induction. Anesthesia will be induced with propofol (1–3 mg/kg), sufentanil (0.3 to 0.4 μg/kg), and rocuronium (0.6 mg/kg) or cisatracurium (0.2 mg/kg). After endotracheal intubation, mechanical ventilation will be performed, with a tidal volume 6–8 ml/kg, a respiratory rate of 12–15/min, a 50–70% fraction of inspired oxygen in the air and fresh gas at a flow rate of 1–2 L/min to maintain the PetCO_2_ between 35 and 40 mmHg.

Before skin incision, a bolus of 0.1–0.2 μg/kg of sufentanil with a beginning infusion rate of 0.1–0.2 μg/kg.min of remifentanil will be given, and the dosage of remifentanil and sufentanil will be adjusted according to the signs of blood pressure and heart rate to maintain between the range of ±15% of baseline. Local anesthetics infiltration in the scalp will be performed at the time of incision. Muscle relaxants can be added intermittently. Crystal or colloid fluid, the urine volume and blood loss will be monitored. Blood transfusion practice guidelines will be resort to once considering blood transfusion [29]. If necessary, vasoactive drugs can be used to maintain the blood pressure and heart rate between the range of ±15% of baseline. Single dose of sufentanil 1 μg/kg, tramadol 100 mg, and ondansetron 8mg will be given to the patients as dura closure. The patients will be extubated after full recovery from anesthesia and transferred to the post-anesthesia care unit (PACU). The patient-controlled intravenous analgesia will be connected to the patients with sufentanil 100 μg and ondansetron 16 mg in 100 ml 0.9% sodium chloride solution, with background dose 2 ml/h. After pressing the pump button to reduce pain, one single 0.5 ml (0.5 μg) will be infused with 15 min locking time.

### Outcomes

The primary outcome is OS (by months) of the patients within 18 months postoperatively. OS is the duration from the day of surgery to the date of death or last follow-up. The assessment of outcomes will be performed by trained research assessors who are blinded to the group allocation. They will assess the OS through telephone interviews with patients or their relatives every 3 months postoperatively.

#### Secondary outcomes include:

1. PFS is the duration from the surgery day to the day of first evidence of progression or death within postoperative 18 months. PFS will be evaluated by using the latest-two MRI tests and clinical evidence according to the RANO (response assessment in neuro-oncology) criteria (Supplementary Table 1) [30].
2. Numerical rating scale (NRS) of pain intensity and sleep quality on postoperative 1 to 3 days will be evaluated. Pain intensity score in NRS is 0 to 10, 0 representing no pain and 10 representing the worst pain imaginable. Sleep quality with the NRS (0 = worst sleep, 10 = best sleep) will be used to evaluate the sleep quality at 8 to 10 a.m. every day postoperatively before discharge.
3. Encephaloedema volume will be calculated from MRI assessed by a radiologist at 3 to 7 days postoperatively.
4. Adverse events or complications will be recorded, including congestive heart failure, arrhythmia, myocardial ischemia or infarction, pulmonary edema, pulmonary embolism, respiratory failure, stroke, encephaloedema, hydrocephalus, pneumocranium, intracranial hematomas or infection, epilepsy, gastrointestinal hemorrhage, liver, and kidney dysfunction (Supplementary Tables 2, 3).
5. The length of hospital stay will be calculated and analyzed.

### Data collection and management

After obtaining informed consent, an independent research assistant will initiate baseline information collection one day before surgery. All personal information will be kept strictly confidential for research purposes only. Basic demographic information, including gender, age, vital signs, body mass index, past medical history, family history, smoking and drinking history, allergy history, operation history or anesthesia history, medication history, age-adjusted Charlson comorbidity index (aCCI, a widely used scoring system to predict outcomes of variety of medical conditions and malignancies, including the age of the patient as a correction variable of the final score of the CCI with the addition of one point for every decade over 40 years), ASA classification, KPS, Glasgow coma scale (GCS) will be collected. Functional classification of tumor (indicating the resectability of tumor) will be evaluated according to the results of preoperative MRI (Supplementary Table 4) [31, 32].

Intraoperative viable include vital sign parameters during anesthesia, total dose or infusion rate of anesthetic agents or other drugs during operation, such as inotropic or vasoactive drugs, mannitol, estimated blood loss, urine volume, the type, and dose of fluid and blood transfusion, duration of surgery and anesthesia. The usage of the analgesia pump (dosage, number of compressions, and effective compressions) will be recorded.

Postoperative medication will be recorded including sedative, analgesia, glucocorticoids, mannitol, and fluid infusion on 1 to 3 days postoperatively. The extent of resection (EOR) will be evaluated by a professional neuro-radiologist according to the results of the MRI within 72 h postoperatively (Supplementary Table 4).

The median follow-up time will be 18 months postoperatively before the study was terminated. The patients and their legal representatives will be told to be followed up by telephone at 3, 6, 9, 12, 15, and 18 months postoperatively. The start points of simultaneous radiotherapy and chemotherapy, patient compliance, adverse events or complications and treatment, the European organization for the research and treatment of cancer on quality of life (EORTCQLQ-C30), and the Karnofsky performance Status (KPS) Scale will be evaluated every 3 months postoperatively. Strict data management will be kept including double data entry and establishing range checks for data values.

### Sample size calculation and statistical analysis

The sample size is estimated by using PASS 2011 software (NCCS LLC). We calculated the sample size based on the primary outcome. A two-sided Log-rank test with an overall sample size of 344 subjects (172 in the intravenous group and 172 in the inhalation group) achieves 80% power at a 0.05 significance level to detect a hazard ratio of 1.66 when the overall survival is 15 vs. 11 months in intravenous and inhalation group, respectively. The study will last for about 42 months, of which subject accrual (entry) occurs in the first 24 months with the proportion dropping out of the two groups being 0.1, respectively.

Analysis will be conducted using SPSS software (version 23.0). The Kolmogorov-Smirnov test will be used to test data normality. Normally distributed continuous variables will be described as the means with standard deviation (SD) and compared by using independent *t* tests. Non-normally distributed variables will be summarized as medians and interquartile range (IQR) and compared by using Mann-Whitney *U* tests. Categorical variables will be described as numbers (*n*) and percentage (%) and compared using chi-square or Fisher’s exact tests.

The Kaplan-Meier method will be used to describe the time-to-event data (OS and PFS), and the log-rank test will be used for comparisons. Univariate and multivariate Cox proportional hazard regressions will be used for survival analysis. Together with the anesthesia type, variables with *P*<0.1 in the univariate analysis will be regarded as candidate variables for the multivariable model. A significance level of *P*<0.05 will be used to indicate statistical significance. The imputation method will be used for dealing with missing data. The impact of missing data will be estimated by sensitivity analysis. Additionally, subgroup analysis will be undertaken to assess the association between anesthesia and the outcome according to the aCCI, KPS, EOR, WHO grade, duration of anesthesia, and whether using chemotherapy or not. No interim analysis will be done and the trial will not early terminate.

The patients receiving reoperation for postoperative complications with different anesthesia methods will be analyzed with the intention-to-treat principle, a secondary per-protocol analysis where patients with protocol deviations are removed. The patients whose postoperative pathological results excluded high-grade glioma will be analyzed with the per-protocol principle. The patients of dropouts, lost follow-up, or end of the study will be treated as censoring in survival analysis.

### Reporting of adverse events

Data monitoring committee will not be set up in the trial for the sample size is not very large. However, an auditing trial will be conducted twice per year and the process will be independent from investigators and the sponsor. All adverse events (AEs) will be closely monitored until a stable situation has been reached. The chief investigator will be informed of any serious AEs and determine the severity and causality of these events. All AEs associated with this study will be recorded and reported to the Ethics Committee as part of the annual report. The chief investigator will be responsible for collecting the details about the causes of AEs, treatment, prognosis, and reporting SAE to the Ethics Committee immediately. The incidence of adverse effects will be described as percentages and frequencies for each group. Chi-square and Fisher’s exact test will be used for comparison between groups. The researcher will have access to the final trial data set.

### Protocol amendment

The chief investigator will be responsible for any decision to amend the protocol. If there is any modification (e.g., changes to eligibility criteria, outcomes, or analysis), the principle investigator will report and gain approval from the China Ethics Committee of Registering Clinical Trials prior to implementation, by communicating with relevant other members (e.g., investigators, participants, registries, journals, and regulators).

## Discussion

This is a randomized controlled trial to compare the effect of intravenous or inhalation anesthesia maintenance on the OS in supratentorial HGG patients of KPS<80. There are some well-known risk factors such as age at diagnosis, KPS, histological type, distant metastasis, and extent of surgical resection for predicting the OS of patients with HG G [31].

Intravenous and inhalation anesthesias are the common anesthesia methods for HGG resection. But they are different in some aspects. Firstly, intravenous anesthesia inhibits the invasion of the tumor cells and host stress response and protects cellular immunity, while inhalation anesthesia has the opposite effect. Secondly, during craniotomy, the mass effect of the tumor and the peritumoral edema always lead to severe intracranial hypertension and brain herniation. As nearly all inhalational agents increase the cerebral blood flow and ICP, propofol has the opposite effect to decrease the ICP and facilitate tumor exposure. Thirdly, intravenous anesthesia promotes postoperative rapid recovery of the patients. Postoperative pain, nausea and vomiting, and shivering appear less frequently with propofol-maintained anesthesia [16, 33, 34]. However, the hemodynamic stability, time to extubation, and early cognitive functions were similar with two anesthetic techniques [35].

In this RCT study, we will fully evaluate the patients before operation, including physical status, neurological function, and imaging results. During the operation, standard anesthesia management, anesthesia depth monitoring, and individualized medication according to the protocol will be conducted by specially trained personnel. After surgery, the patients will be followed up regularly by designated personnel for the recovery, adverse events, complications, treatment, PFS, and OS.

Although the intervention of our study is intravenous or inhalation anesthesia, inhalational anesthetics will not be used for induction, as it is not routine in clinical practice. So propofol induction will be used in both groups. We regard the effect of a single bolus of propofol as minimal and short, which will be eliminated completely before surgical incision.

We use the survival time to calculate the sample size. As the median OS was 15 vs.11 months in TIVA and inhalation group, with a hazard ratio of 1.66, the calculated sample size is 344, which is more credible in survival analysis.

Our study will optimize the ideal anesthesia regimen for patients undergoing brain tumor resections, not only from the point of view on perioperative anesthesia and analgesia, but also from the point of view on clinical outcomes of overall survival in HGG patients.

## Trial status

The trial was registered at ClinicalTrials.gov on 29 April 2016 (identifier NCT02756312) and the last update time of the trial is 9 Sep 2020. The study was approved by the Ethics Review Committee of China Registered Clinical Trials (Ethics Review No. ChiECRCT-20200049). The trial has not yet recruited any participants till now. The anticipated completion date will be in December 2022.

## Supplementary Information

**Additional file 1: Supplementary Table 1.** Rano criteria for tumor progression. **Supplementary Table 2.** The diagnosis criterion of complications. **Supplementary Table 3.** Muscle strength grade. **Supplementary Table 4.** Brain tumors assigned grades based on functional location. **Supplementary Table 5.** The extent of resection (EOR) of the tumor.

## Data Availability

This study will be conserved in a secure repository at Beijing Tiantan Hospital. The data sets will be available from the chief investigator upon reasonable request.

## Abbreviations

HGG: High-grade glioma
OS: Overall survival
PFS: Progression-free survival
NRS: The numerical rating scale
WHO: World Health Organization
ICP: Intracranial pressure
NK: Natural killer
HIF: Hypoxia-inducible factor
NLR: Neutrophil–lymphocyte ratio
PLR: Platelet–lymphocyte ratio
KPS: Karnofsky Performance Status
ASA: American Society of Anesthesiologists
MRI: Magnetic radiology imaging
SAE: Serious adverse event
BIS: Bispectral index
PetCO_2_: End-tidal carbon dioxide partial pressure
MAC: Minimal alveolar concentration
PACU: Post-anesthesia care unit
GCS: Glasgow coma scale
EORTCQLQ-C30: The European organization for the research and treatment of cancer on quality of life
Rano: Response assessment in neuro-oncology
