# Supplementary material for: Effect of anesthesia on the outcome of high-grade glioma patients undergoing supratentorial resection: study protocol for a pragmatic randomized controlled trial
Source: Trials. 2022 Sep 27;23:816. doi: 10.1186/s13063-022-06716-9 (PMC9513932; doi:10.1186/s13063-022-06716-9)
Supplement: Supplementary file 1 — Additional file 1: Supplementary Table 1. Rano criteria for tumor progression. Supplementary Table 2. The diagnosis criterion of complications. Supplementary Table 3. Muscle strength grade. Supplementary Table 4. Brain tumors assigned grades based on functional location. Supplementary Table 5. The extent of resection (EOR) of the tumor. [file 13063_2022_6716_MOESM1_ESM.docx]

| Supplementary Table 1. Rano criteria for tumor progression. | |
| --- | --- |
|  | More than 25% increase in sum of the products of perpendicular diameters of enhancing lesions compared with postoperative baseline measurements, despite stable or increasing doses of corticosteroids; |
|  | Significant increase in T2/FLAIR non-enhancing lesion on stable or increasing doses of corticosteroids compared with baseline scan； |
|  | Any new lesion; |
|  | Clear clinical deterioration not attributable to other causes apart from the tumor (eg, seizures, medication adverse effects, complications of therapy, cerebrovascular events, infection, and so on) or changes in corticosteroid dose; |
|  | Failure to return for evaluation as a result of death or deteriorating condition; |
|  | Clear progression of non-measurable disease. |

Abbreviations: FLAIR, fluid attenuated inversion recovery

**Supplementary Table 2. The diagnosis criterion of complications.**

| **System** | **disease** | **Definition** |
| --- | --- | --- |
| Cardiac system | Myocardial infarction | Typical increase in troponin level, or rapid rise and fall in CK-MB, accompanied by one of the following: symptoms of myocardial ischemia; pathological Q wave; EKG with ischemic change; coronary intervention; and / or echocardiography or imaging with new or suspected new abnormal wall motion. |
|  | Cardiac arrest | The successful resuscitation of a definite or suspected ventricular fibrillation or sustained ventricular tachycardia or cardiac arrest. |
|  | Arrhythmias | requiring drug treatment: diagnosis by bedside EKG. |
| Respiratory system | Pneumonia | High fever, cough and expectoration were accompanied by positive chest X-ray or CT findings or sputum culture. |
|  | Respiratory failure | The results of arterial blood gas analysis showed that hypoxemia and hypercapnia (PaO_2_ < 60mmHg and / or PaCO_2_ > 50mmHg) needed to establish airway urgently for ventilation support treatment. |
| Surgical site infection (SSL) | Incision infection | The wound is suppurated or secretion cultured positive within 30 days postoperatively. Or the wound is red, swollen, or painful, and reopened by the surgeon suspecting of infection. |
|  | Intracranial infection | CSF culture positive, or brain abscess confirmed by the second operation, or CSF routine examination positive (WBC > 100 × 10^6^ / L + proportion of polynuclear white blood cells > 50% + glucose concentration < 2.5mmol / L), at the same time, there were suspicious manifestations of intracranial infection such as fever (>38 ℃, lasting for 3 days), positive meningeal stimulation sign or change of consciousness within 90 days after neurosurgery |
| Digestive | Gastrointestinal bleeding | Patients have hematemesis or coffee like substances, black stool or stool with blood postoperatively, which is diagnosed as gastrointestinal bleeding by physicians and needs protective intervention or treatment. |
| Coagulation | Deep vein thrombosis(DVT) | DVT in the lower extremity was confirmed by the results of Doppler ultrasound. |
|  | Pulmonary embolism | The dyspnea and hypoxemia after pulmonary embolism were diagnosed by respiratory physician. |
| Nervous system | Intracranial hematomas | CT showed high density or hematocele in the brain, compressed the surrounding brain tissue, or the drainage tube placed in the brain continued to drain the hemorrhagic fluid. |
|  | Ischemic stroke | MRI scan of shows new cerebral infarction. |
|  | Encephaloedema | Patients have intracranial hypertension or brain hernia, or suddenly deteriorate of the state of consciousness by CT or MRI reporting encephaloedema. |
|  | Hydrocephalus | When the state of consciousness suddenly worsen or the symptoms of intracranial hypertension appears, CT showed that the lateral ventricles were obviously dilated postoperatively: ① enlargement of the upper and lateral frontal horn; ② temporal horn enlargement; ③ low density around the ventricles. |
|  | Status epilepticus | Refers to the status of epilepticus if the consciousness is not fully recovered and the seizure is frequent or lasts for more than 30 minutes, which means the patient has frequent attacks and can't stop by himself or although the attack is not frequent, the patient's consciousness has not turned clear. |
|  | Hemiplegia | Limited limbs activity or muscle strength (Table 4) less than Grade III. |

| Supplementary Table 3. Muscle strength grade. | |
| --- | --- |
| Grades Definition | |
| Grade 0 | no contraction (complete paralysis) |
| Grade I | slight contraction of muscle, but unable to move joints (close to complete paralysis) |
| Grade II | muscle contraction can drive joints to move horizontally, but unable to resist gravity (severe paralysis) |
| Grade III | able to resist the gravitational movement of joints, but unable to resist resistance (mild paralysis) |
| Grade IV | able to resist the gravitational movement of limbs and resist a certain strength of resistance (close to normal) |
| Grade V | able to resist the strong resistance movement of limbs (normal) |

| Supplementary Table 4. Brain tumors assigned grades based on functional location. | |
| --- | --- |
| Grades | **Tumor location** |
| Grade I | non-eloquent brain, frontal or temporal lesion, right parieto-occipital or cerebellar hemispheric lesions |
| Grade II | near motor, sensory or calcarine cortex or near the speech center or corpus callosum, |
| Grade III | motor/sensory cortex, visual center, speech center, internal capsule, basal ganglia or hypothalamus/thalamus |

| Supplementary Table 5. The extent of resection (EOR) of the tumor. | | |
| --- | --- | --- |
| Extent of resection (EOR) | **Residual enhancement** | **Resection proportion** |
| Gross total resection (GTR) | no residual enhancement | ≧99% |
| Near total resection (NTR) | only rim enhancement | 95-99% |
| Subtotal resection (STR) | residual nodular enhancement | 90-95% |
| Partial resection (PR) | residual mass enhancement | <90% |
